# Combining SGLT2 Inhibition With a Thiazolidinedione Additively Attenuate the Very Early Phase of Diabetic Nephropathy Progression in Type 2 Diabetes Mellitus

**DOI:** 10.3389/fendo.2018.00412

**Published:** 2018-07-19

**Authors:** Eugene Han, Eugene Shin, Gyuri Kim, Ji-Yeon Lee, Yong-ho Lee, Byung-Wan Lee, Eun Seok Kang, Bong-Soo Cha

**Affiliations:** ^1^Division of Endocrinology and Metabolism, Department of Internal Medicine, Keimyung University School of Medicine, Daegu, South Korea; ^2^Graduate School, Yonsei University College of Medicine, Seoul, South Korea; ^3^Institute of Endocrine Research, Yonsei University College of Medicine, Seoul, South Korea; ^4^Division of Endocrinology and Metabolism, Department of Internal Medicine, Yonsei University College of Medicine, Seoul, South Korea

**Keywords:** sodium glucose co-transporter 2 inhibitor, thiazolidinedione, diabetic nephropathy, type 2 diabetes, db/db mice

## Abstract

Although both sodium glucose co-transporter 2 inhibition by dapagliflozin and thiazolidinedione, pioglitazone have glucose-lowering and anti-inflammatory effects, the therapeutic efficacy of their combination on diabetic nephropathy has not been investigated. 9-week-old male db/db mice were randomly assigned to 4 groups and administrated with (1) vehicle, (2) dapagliflozin, (3) pioglitazone, or (4) dapagliflozin and pioglitazone combination. Human proximal tubule (HK-2) cells were treated with glucose or palmitate acid in the presence of medium, dapagliflozin, pioglitazone, or both. Glomerular tuft area and mesangial expansion of the kidney more reduced in the combination group compared to control and single therapy groups. Podocyte foot process width and glomerular basement membrane thickness decreased regardless of treatment, while the combination group showed the slowest renal hypertrophy progression (*P* < 0.05). The combination treatment decreased MCP-1, type I and IV collagen expression in the renal cortex. Only the combination treatment decreased the expression of angiotensinogen, IL-6, and TGF-β while it enhanced HK-2 cell survival (all *P* < 0.05). In conclusion, dapagliflozin and pioglitazone preserved renal function, and combination therapy showed the greatest benefit. These findings suggest that the combination therapy of dapagliflozin with pioglitazone is more effective than the single therapy for preventing the progression of nephropathy in patients with type 2 diabetes.

## Introduction

Sodium glucose co-transporter 2 (SGLT2) inhibitors block glucose reabsorption in the proximal tubules, and consequently stimulate glucose excretion in the urine (1). Dapagliflozin is a highly selective and first-in-class SGLT2 inhibitor, which has many favorable effects on glucose lowering and body weight loss in clinical studies (2, 3). The result of preclinical and animal studies demonstrated other beneficial effects of this SGLT2 inhibitor such as improved glucose homeostasis (4), preserved pancreatic islet cell function (5, 6), enhanced muscle insulin sensitivity (7), and attenuated hepatic steatosis (8).

Pioglitazone is in the class of thiazolidinedione (TZD), which plays essential roles in improving glucose tolerance and insulin sensitivity (9). Although TZD has a protective effect against cardiovascular disease and inflammation (10, 11), this class of drug induces fluid retention and edema, and aggravates congestive heart failure because of increased sodium reabsorption (12).

Because of the sodium excreting effect of dapagliflozin, it is plausible that dapagliflozin can prevent the peripheral edema that may be induced by pioglitazone treatment. Reducing excess glucose combined with improving insulin sensitivity could be an ideal combination for obese patients with type 2 diabetes (T2D). However, there is limited information on the combination therapy of SGLT2 inhibitor and TZD in diabetic nephropathy. We hypothesized that the combination therapy could have a synergistic effect or at least have an additive effect on preventing diabetic nephropathy in type 2 diabetes model. Therefore, the aim of the present study was to investigate the therapeutic effect of combination therapy in an animal model to support the experimental rationale for the combination therapy of pioglitazone and dapagliflozin.

## Materials and methods

### Animals and study design

Eight-week-old male db/db mice were purchased from Jackson Laboratories (Bar Harbor, ME, USA). After 1 week of acclimatization, mice were divided into four groups: (1) vehicle control (phosphate-buffered saline [PBS; Amresco, Solon, OH, USA] solution) (*n* = 5), (2) 30 mg/kg/day pioglitazone (*n* = 8), (3) 2 mg/kg/day dapagliflozin (*n* = 8), or (4) a combination of 2 mg/kg/day dapagliflozin plus 30 mg/kg/day pioglitazone (*n* = 7). Vehicle or drugs were administrated daily by oral gavage for 9 weeks. All animal studies were approved by the Animal Care and Use Committee of the Yonsei University College of Medicine.

### Biochemical measurements

Blood samples for random glucose measurements were obtained via tail tip vein and glucose concentrations were determined with a glucose analyzer (AGM-4100; Allmedicus, Anyang, Korea). On week 8, spot urine was obtained as previously explained (13), and stored at −80°C for analysis. Urinary creatinine was determined using an autoanalyzer (Molecular Devices, Sunnyvale, CA, USA) and urinary albumin concentrations were measured using a commercially available enzyme-linked immunosorbent assay (ELISA) kit (Abcam, Cambridge, Cambridge, UK) according to the manufacturer's protocol. At week 9, an oral glucose tolerance test was performed following a 6-h fast (14), and blood samples were taken via tail prior to (0 min) and following an oral glucose bolus (1 g/kg) at 30, 60, 90, and 120 min to measure plasma glucose concentration. At the end of treatment, general anesthesia was induced via inhalation of 5% isoflurane. Blood samples were obtained by left ventricular puncture and stored at −70°C for subsequent analyses, were centrifuged at 5,000 × g for 15 min at 4°C. Plasma concentrations of triglycerides (TG; BioVision, Milpitas, CA, USA) and free fatty acid (FFA; BioAssay Systems, Hayward, CA, USA) were measured using a colorimetric method according to the manufacturer's protocols. Analysis of blood urea nitrogen (BUN) concentration was performed in the Eone Reference Laboratory (http://www.eonelab.co.kr, Republic of Korea).

### Tissue collection and histological analysis

Paraffin-embedded kidney tissues were cut into 4 μm thick sections and stained with Hematoxylin and Eosin (H&E), Periodic Acid-Schiff (PAS), and Masson's trichrome stain. All tissue sections were examined using a BX40 microscope (OL-BX40, Olympus, Tokyo, Japan). Mesangial expansion and glomerular hypertrophy were assessed in a minimum of 15 glomeruli per mouse kidney. The tissue sections were magnified at ×400 and the diameter of the glomerular tuft and PAS-positive areas were quantified from the glomerulus cut in a plane along the vascular pole. For transmission electron microscopic analysis, kidney cortical samples were fixed with a solution containing 3% glutaraldehyde plus 2% paraformaldehyde in 0.1 mol/L phosphate buffer (pH 7.4), followed by 1% osmium tetroxide. After dehydration, thin sections were stained with uranyl acetate and lead citrate for observation under a JEM 1011CX electron microscope (JEOL, USA, Inc.). Histological images were analyzed using ImageJ software (NIH Image, Bethesda, MD, USA) for quantifying mesangial expansion and glomerular hypertrophy.

### *In situ* cell death detection

To investigate the cell death of kidney tubule, Terminal deoxynucleotidyl transferase (TdT)-mediated digoxigenin-dUTP nick end labeling (TUNEL) was performed on fixed tissue sections using a commercially available kit (TACS® 2TdT DAB kit, Trevigen, Gaithersburg, MD, USA) according to the manufacturer's instructions. Twenty randomly selected areas per mouse kidney were evaluated under high power magnification (×400).

### Real-time PCR

The kidney RNA was prepared using Trizol reagent (Thermo Fisher, Grand Island, NY, USA) according to the manufacturer's instructions. Reverse transcription was performed using the high capacity complementary DNA transcription kit (Applied Biosystems, Foster City, CA, USA) by real time polymerase chain reaction (RT-PCR) using the SYBR Green Master Mix (Thermo Fisher, Grand Island, NY, USA). Expression of transforming growth factor (TGF)-β, monocyte chemoattractant protein (MCP)-1, type I and type IV collagens, renin, interleukin (IL)-6, and angiotensinogen (AGT) was normalized to the reference gene, glyceraldehyde-3-phosphate dehydrogenase (GAPDH) (Supplementary Table 1).

### Cell culture

An immortalized human proximal tubule epithelial cell, HK-2 was maintained in Dulbeco's Modified Eagle's Media (DMEM) containing 25 mM D-glucose supplemented with 10% fetal bovine serum, penicillin (100 U/mL), and streptomycin (100 μg/mL) (Thermo Fisher, Grand Island, NY, USA) (15). When cell confluency reached to 80% confluence, cells were exposed to (1) 5.5 mM glucose, (2) 50 mM glucose, (3) 50 mM glucose plus 0.3 mM palmitic acid (Sigma-Aldrich, Saint Louis, MO, USA), (4) 10 μM pioglitazone plus (3) medium, (5) 10 μM dapagliflozin plus (3) medium, or (6) 10 μM pioglitazone and 10 μM dapagliflozin co-treatment in (3) medium, for 24 h then harvested (*n* = 5).

### Cell viability assay

It was reported that palmitic acid can induce cell death in HK-2 cells (16–19). To test the effect of dapagliflozin and pioglitazone on palmitic acid and high glucose induced HK-2 cell death, Cell viability was measured by the MTT (3-(4-5-Dimethylthiazol-2-yl-2,5-diphenyltetrazolium bromide) assay. HK-2 cells were seeded into in 96-well plates and incubated overnight to allow the cells to adhere and, were exposed to same manner to the condition used for cell culture study [(1) 5.5 mM glucose, (2) 50 mM glucose, (3) 50 mM glucose plus 0.3 mM palmitic acid, (4) 10 μM pioglitazone plus (3) medium, (5) 10 μM dapagliflozin plus (3) medium, or (6) 10 μM pioglitazone and 10 μM dapagliflozin co-treatment in (3) medium]. At the end of exposure, 40 μMl of MTT solution (2 mg/ml) was added and the cells were then incubated with WST-8 (Dojindo Laboratories, Kumamoto, Kumamoto, Japan) solution at 37°C for 1 h. Cells were solubilized with 150 μl of DMSO and absorbance was quantified in 450 nm using a microplate reader (VersaMax ELISA Microplate Reader, Molecular Devices, Sunnyvale, CA, USA). The cell viability index was calculated as the percentage of control group which was assumed to be 100%.

### Western blot

Membrane and cytoplasmic proteins were extracted from cultured HK-2 cell using the Mem-PER Plus Membrane Protein Extraction Kit (Pierce Biotechnology, Rockford, IL, USA) and measured suing the bicinchoninic acid assay (Pierce Biotechnology) according to the manufacturer's instructions. Equal amounts of protein (30 μg/well) were resolved by sodium dodecyl sulfate polyacrylamide gel electrophoresis and analyzed by western blot using specific antibodies against SGLT2 (cat. #37296, Abcam, Bristol, UK) and β-Actin (cat. #47778, Santa Cruz, Dallas, TX, USA). For analysis of the western blot images were analyzed using ImageJ software (NIH Image, Bethesda, MD, USA) for quantifying mesangial expansion and glomerular hypertrophy.

### Statistical analyses

Data were expressed as the mean ± standard error of mean (SEM). All statistical analyses were conducted using IBM SPSS version 23.0 for Windows (IBM Corp., Armonk, NY, USA). Statistical comparisons between groups for body weight and random blood glucose were performed using two-way analysis of variance (ANOVA) for repeated measurements (RM) followed by Tukey's *post-hoc* test. For the oral glucose tolerance test, area under curve (AUC) was calculated and analyzed by 2-way ANOVA. For the data from the experiments with HK-2 cell, one-way ANOVA was followed by the Dunnett *post-hoc* test. For other comparisons between groups were performed using one-way ANOVA, followed by the Tukey's *post-hoc* test; *P* < 0.05 was considered statistically significant.

## Results

### Physical and biochemical characteristics of db/db mice

**Figure 1:**
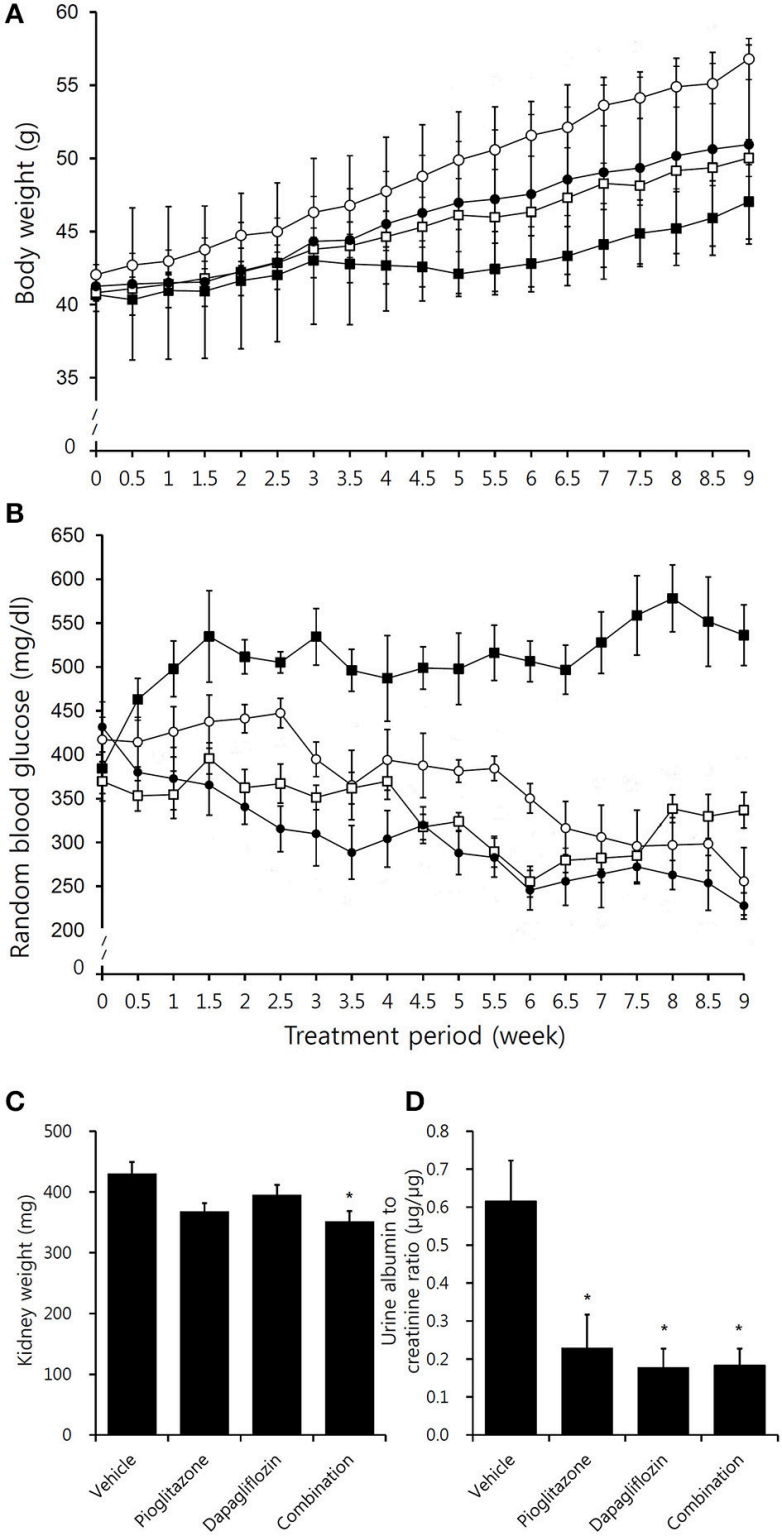
Effects of pioglitazone, dapagliflozin and combination on body weight, blood glucose, kidney weight, and albuminuria. Graph depicting **(A)** body weight and **(B)** random blood glucose concentration in vehicle (black square, ■), pioglitazone (30 mg/kg/day, white circle, ◦), dapagliflozin (1 mg/kg/day,white square □), and combination (30 mg/kg/day of pioglitazone and 1 mg/kg/day of dapagliflozin, black circle, •) during 9 week of study period. **(C)** Measurement of both kidney weight after 9 weeks treatment in vehicle (PBS), pioglitazone (30 mg/kg/day), dapagliflozin (1 mg/kg/day), and combination (30 mg/kg/day of pioglitazone and 1 mg/kg/day of dapagliflozin). **(D)** Measurements of urine albumin to creatinine ratio after 8 weeks treatment in vehicle (PBS), pioglitazone (30 mg/kg/day), dapagliflozin (1 mg/kg/day), and combination (30 mg/kg/day of pioglitazone and 1 mg/kg/day of dapagliflozin). Data are means ± SEM (vehicle *n* = 5, monotherapy *n* = 8, combination therapy *n* = 7). *P < 0.05 vs. vehicle by one-way ANOVA and Tukey's *post-hoc* test.

The body weights of all mice gradually increased during the study period, and the pioglitazone group had significantly greater weight than the other groups beginning at the 4th week of treatment (Figure 1A). The *P* value for interaction on body weight was 0.041, and dapagliflozin monotherapy treated mice weighed less than vehicle treated group (*P* = 0.036 overall independent of time). The amount of food consumption was not significantly different between the groups (*P* = 0.994). The combination treatment group experienced the greatest efficacy with 95.3% reduction from baseline (*P* = 0.002 compared to vehicle) (Figure 1B) (*P* interaction <0.001), and showed the most reduced AUC (Supplementary Figures 1A,B) (*P* interaction = 0.032). Similarly, plasma TG and FFA concentrations were decreased in treatment groups compared to vehicle-treated mice (Supplementary Figures 2A,B). The concentration of BUN was similar across the groups (*P* interaction = 0.846). In conclusion, combination treated group showed the greatest glucose reduction with insignificant difference in body weight and lipid concentration compared to monotherapy groups.

### Renal morphology and albuminuria

Compared to the vehicle-treated group, kidney weights were lowest in the combination group (Figure 1C). Urine albumin to creatinine ratio (UACR) was reduced in all treatment arms, however, no significant difference in UACR was observed between the treatment groups (Figure 1D).

**Figure 2:**
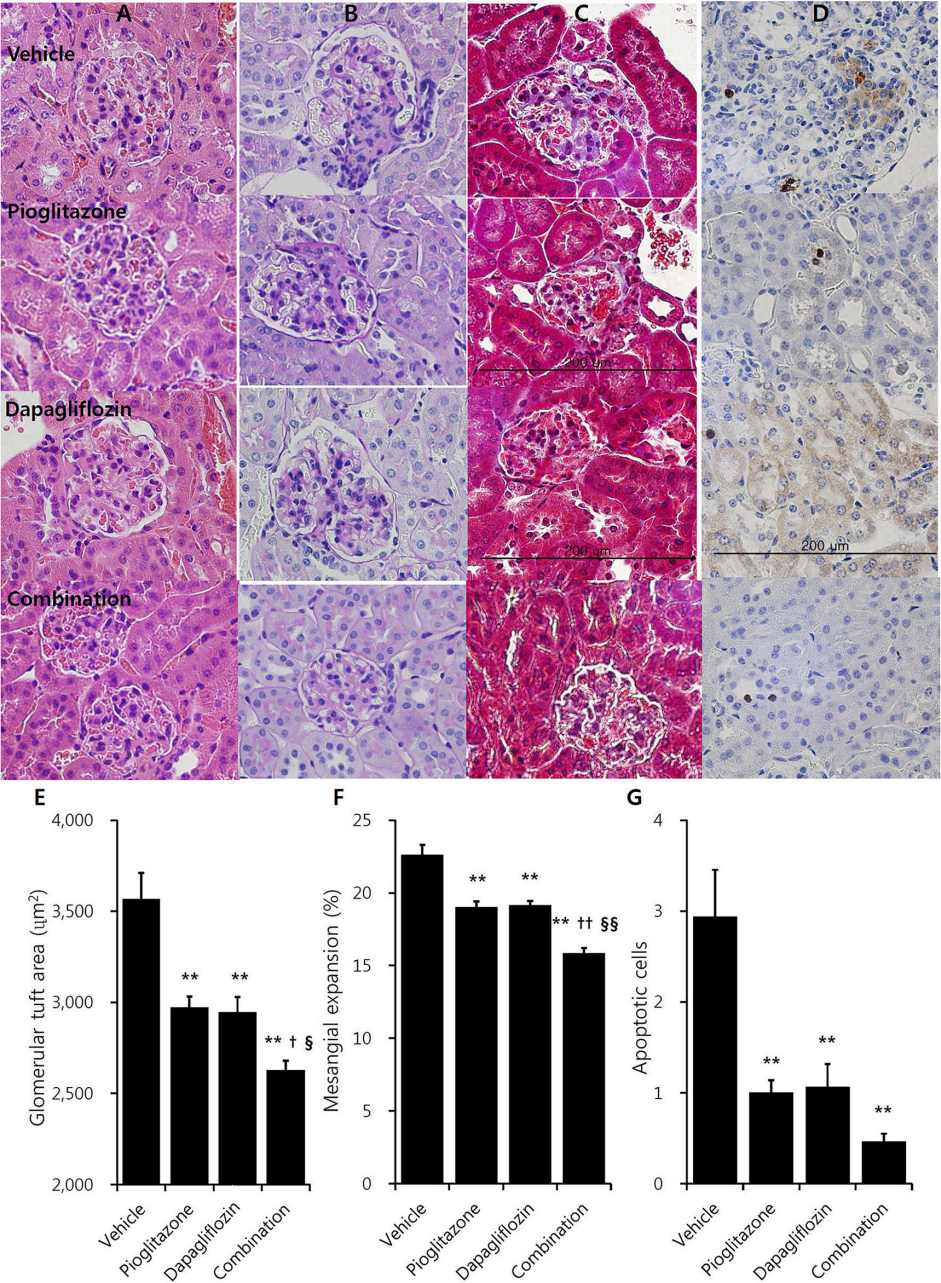
Effects of pioglitazone, dapagliflozin and combination on kidney morphology in immunohistochemistry studies. Renal glomerulus and tubules of 9 weeks treatment in vehicle (PBS), pioglitazone (30 mg/kg/day), dapagliflozin (1 mg/kg/day), and combination (30 mg/kg/day of pioglitazone and 1 mg/kg/day of dapagliflozin). **(A)** Hematoxylin and Eosin (H&E) stain, **(B)** Periodic acid-Schiff (PAS) stain, **(C)** Masson's trichrome stain, **(D)** Terminal deoxynucleotidyl transferase dUTP nick end labeling (TUNEL) stain (×400, bar presents 200 μm). Quantification of **(E)** glomerular tuft area, and **(F)** mesangial expansion in PAS stain section minimum of 15 glomeruli per mouse kidney under high power magnification (×400). **(G)** Counting apoptotic tubular cell in TUNEL stain minimum of 20 randomly selected areas per mouse kidney under high power magnification (×400). Data are means ± SEM (vehicle *n* = 5, monotherapy *n* = 8, combination therapy *n* = 7). **P < 0.001 vs. vehicle, ^†^*P* < 0.05 vs. pioglitazone, ^††^*P* < 0.001 vs. pioglitazone, ^§^*P* < 0.05 vs. dapagliflozin, ^§§^*P* < 0.001 vs. dapagliflozin by one-way ANOVA and Tukey's *post hoc* test.

In immunohistochemistry studies, increased glomerulus size and tubuloglomerular fibrosis were observed in vehicle mice, which were attenuated in the treatment groups (Figures 2A–D). Pioglitazone and dapagliflozin monotherapy groups showed attenuated glomerular hypertrophy, and both monotherapy groups showed comparable glomerular tuft size (2971.1 ± 62.0 μm^2^ for pioglitazone and 2945.2 ± 84.7 μm^2^ for dapagliflozin) (Figure 2E). The combination therapy group showed the greatest reduction in glomerular tuft area (26.4% reduction compared to vehicle, *P* < 0.001). Similarly, the mesangial expansion ratio was lower in the treatment groups, and was lowest in the combination arm (30.0% reduction compared to vehicle, *P* < 0.001) (Figure 2F). Along with these morphologic changes, TUNEL staining demonstrated more cell death in the kidney of vehicle mice, which was lower in the pioglitazone and dapagliflozin groups (Figures 2D,G). With respect to the three treatment arms, mice treated with the combination showed the lowest number of apoptotic cells in the kidney.

**Figure 3:**
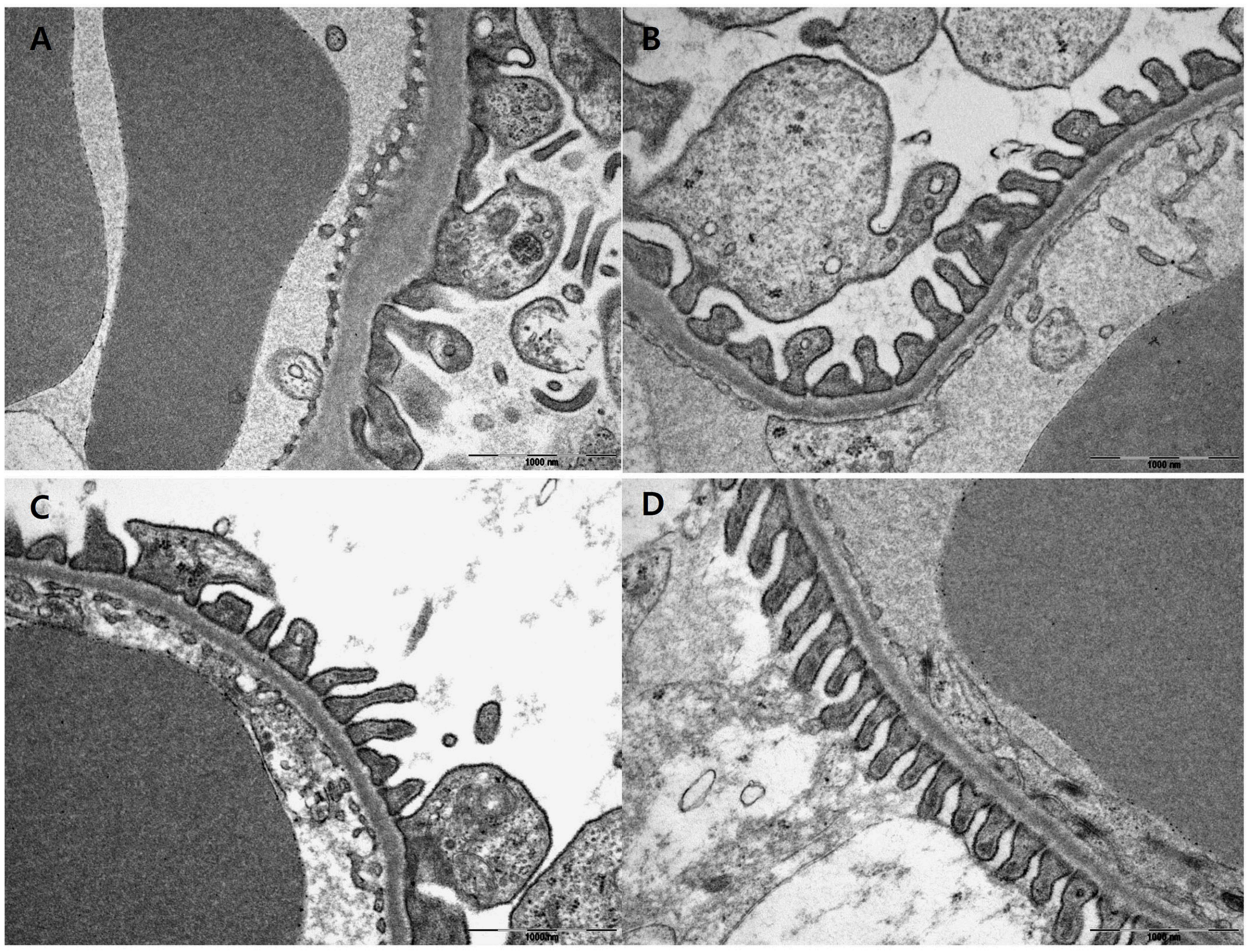
Effects of pioglitazone, dapagliflozin and combination on glomerulus morphology by electron microscope. Glomerulus of 9 weeks treatment in vehicle (PBS, **A**), pioglitazone (30 mg/kg/day, **B**), dapagliflozin (1 mg/kg/day, **C**), and combination (30 mg/kg/day of pioglitazone and 1 mg/kg/day of dapagliflozin, **D**). Glomerulus of each treated group ×30,000 magnification.

**Figure 4:**
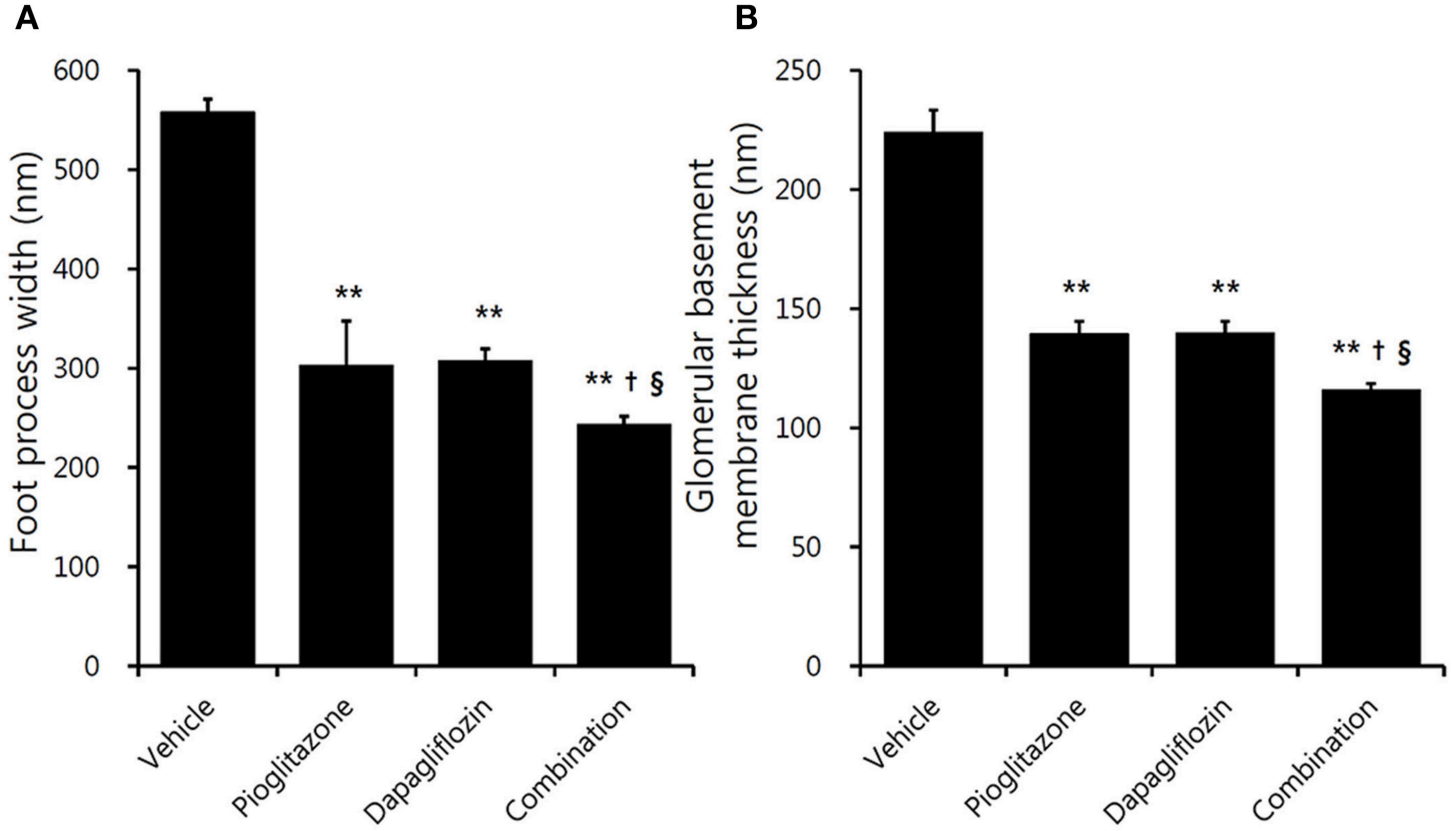
Effects of pioglitazone, dapagliflozin and combination on glomerulus foot process and basement membrane. Quantification of **(A)** foot process width, and **(B)** glomerular basement membrane thickness under high power magnification (×30,000). Data are means ± SEM (*n* = 5–8). **P < 0.001 vs. vehicle, ^†^*P* < 0.05 vs. pioglitazone, ^§^*P* < 0.05 vs. dapagliflozin by one-way ANOVA and Tukey's *post-hoc* test.

Electron microscopic examination showed increased irregular thickening of the glomerular basement membrane (GBM) and foot process effacements on glomeruli in the vehicle mice (Figure 3A). These morphologic changes were attenuated in all treatment groups (Figures 3B–D). Podocyte foot process width was reduced by 45.7 and 44.9%, and GBM thickness was decreased by 37.8 and 37.7% for the pioglitazone and dapagliflozin monotherapy groups, respectively (all *P* < 0.001 compared to vehicle). The combination-treated group showed the best preservation of glomerular morphology with 56.6% reduction in podocyte foot process width and 48.3% reduction in GBM thickness compared to vehicle. In addition, compared to the monotherapy groups, combination treatment resulted in better glomerular structures (GBM thickness *P* = 0.038 compared to pioglitazone, *P* = 0.020 compared to dapagliflozin; foot process width *P* = 0.033 compared to pioglitazone, *P* = 0.027 compared to dapagliflozin) (Figure 4A,B). In conclusion, combination treated group showed the greatest preserved renal morphology change compared to monotherapy and vehicle groups.

### Inflammatory, profibrotic, and renin-angiotensin system-related gene expression

**Figure 5:**
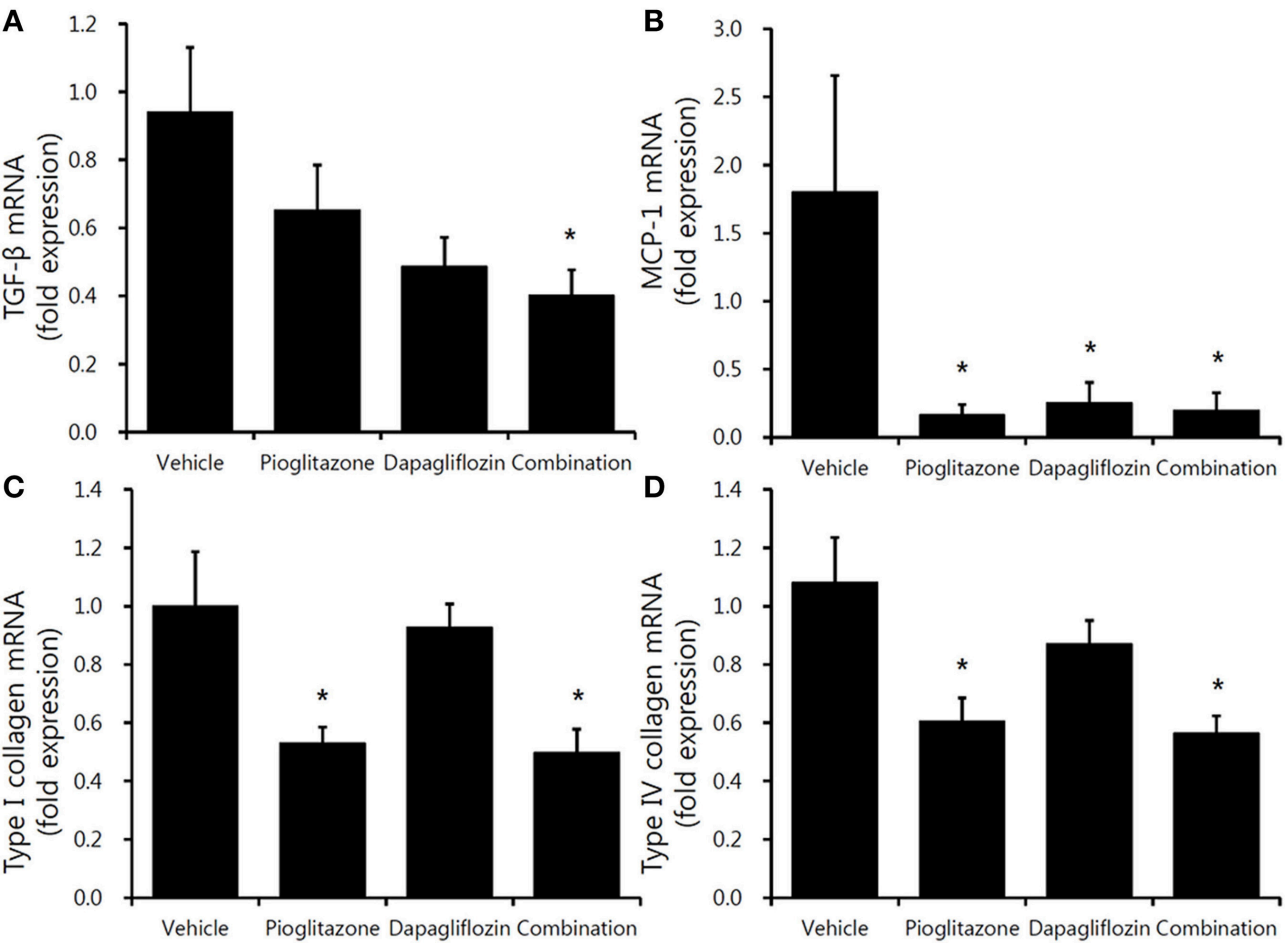
Comparison of inflammatory and profibrotic gene expression in mouse renal cortex. Real-time PCR for 9-week vehicle (PBS)-, pioglitazone (30 mg/kg/day)-, dapagliflozin (1 mg/kg/day)-, and combination (30 mg/kg/day of pioglitazone and 1 mg/kg/day of dapagliflozin)-treated mice' renal cortex. **(A)** TGF-β (encoding transforming growth factor β), **(B)** MCP-1 (encoding monocyte chemoattractant protein-1), **(C)** type 1 collagen, **(D)** type IV collagen. Data are means ± SEM (vehicle *n* = 5, monotherapy *n* = 8, combination therapy *n* = 7). *P < 0.05 vs. vehicle by one-way ANOVA and Tukey's *post-hoc* test.

Decreased trends in inflammatory gene expression were observed in the three treatment groups. TGF-β mRNA level was significantly decreased only in the combination-treated mice (Figure 5A). Regarding MCP-1, all treatment groups had decreased expression compared to vehicle; however, there was no significant differences between the groups (Figure 5B). The fibrosis markers, type I and type IV collagen, were decreased in the pioglitazone monotherapy and combination groups (Figure 5C,D) (all *P* < 0.05).

**Figure 6:**
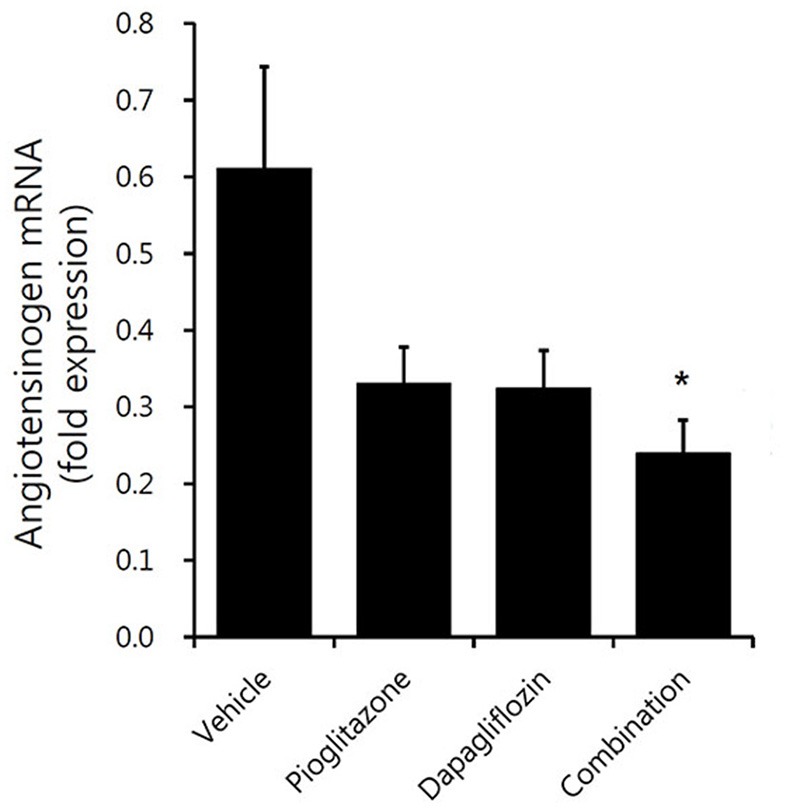
Comparison of angiotensinogen expression in mouse renal cortex. Real-time PCR for 9-week vehicle (PBS)-, pioglitazone (30 mg/kg/day)-, dapagliflozin (1 mg/kg/day)-, and combination (30 mg/kg/day of pioglitazone and 1 mg/kg/day of dapagliflozin)-treated mice' renal cortex. Data are means ± SEM (vehicle *n* = 5, monotherapy *n* = 8, combination therapy *n* = 7). *P < 0.05 vs. vehicle by one-way ANOVA and Tukey's *post-hoc* test.

The renal renin-angiotensin system (RAS) activity tended to decrease in the treatment groups (Figure 6). Dapagliflozin and pioglitazone-treated mice showed lower AGT expression compared to vehicle-treated mice; however, only the combination group had reduced AGT expression (*P* = 0.008). In conclusion, the combination treated group showed the greatest reduction in renal markers of inflammation and fibrosis compared to both the monotherapy and vehicle groups.

### Effect of dapagliflozin and pioglitazone treatments on HK-2 cells

As vehicle db/db mice showed increased blood glucose and lipid concentrations compared to treated mice, we evaluated high glucose and high lipid-induced inflammation and RAS activation and the direct renoprotective effect of dapagliflozin, pioglitazone, and combination therapy in *in vitro* studies. Compared to cells in normal glucose, the TGF-β/GAPDH mRNA ratios were 6-fold higher in tubular cells exposed to high glucose and palmitate medium (*P* < 0.05) (Supplementary Figure 3A). This increase in TGF-β mRNA expression was attenuated by single or combination treatment (all *P* < 0.05). The levels MCP-1 protein in conditioned culture medium showed a similar pattern to the TGF-β mRNA expression (Supplementary Figure 3B). The level of IL-6 protein was significantly decreased only in the combination-treated mice (Supplementary Figure 3C). The renin expression was not significantly different across the groups (Supplementary Figure 3D). However, the increase in AGT was significantly attenuated with all treated mice group (Supplementary Figure 3E). Supplementary Figure 3F depicts the trend that showed cell survival recovery in the treatment groups; monotherapy and combination treatment had statistically significant recovery. Moreover, the concentration of SGLT2 protein in the HK-2 membrane fraction was not different between the groups (Supplementary Figure 4; *P* = 0.002 for pioglitazone, *P* = 0.001 for combination). In conclusion, combination treated group showed the greatest reduction in IL-6 expression with insignificant difference in MCP-1, TGF-β, AGT, and cell recovery compared to monotherapy groups.

## Discussion

In the present study, we tested the hypothesis that dapagliflozin and pioglitazone combination therapy would prevent T2D-related renal injury and examined its effects on metabolic parameters in a db/db mouse model. The results showed that pathological changes in renal cortex, increased albuminuria, and upregulated expression of fibrotic and RAS-related genes in the kidney were ameliorated in the dapagliflozin, pioglitazone, and combination treatment groups. Furthermore, the most attenuation of glomerular hypertrophy, amelioration of fibrosis, and AGT gene expression was observed in the combination group. We also demonstrated that dapagliflozin, pioglitazone, and combination therapy resulted in reduced inflammatory and RAS-related gene expression in the HK-2 cell experiments.

Classically, the anti-inflammatory mechanism of TZD on diabetic nephropathy has been well established (20–22). An *in vitro* study of mesangial cells, pioglitazone attenuated high glucose-induced MCP-1 synthesis, NF-kB activation, and collagen synthesis (23). In addition, increased antioxidant enzyme induction (Cu-Zn SOD, GSH-Px) in the kidney was ameliorated in a TZD-treated type 1 diabetes animal model, without the glucose-lowering effect, which suggested that TZD has an independent renoprotective effect based on reactive oxygen species inhibition (24). In our study, upregulated expression of TGF-β, type I and type IV collagens was ameliorated in the pioglitazone monotherapy group. Along with TZD, recent studies support the evidence that SGLT2 inhibitors have a beneficial effect on diabetic nephropathy. SGLT2 in the proximal tubules reabsorb the majority of glucose in the kidney; thus, inhibition of SGLT2 can lower glucose concentration as well as diabetes-related complications (25, 26). In a 12-week study, dapagliflozin fed to male *db/db* mice resulted in decreased macrophage infiltration by improvement of hyperglycemia in a dose-dependent manner (27). Similarly, treatment of *db/db* mice with another SGLT2 inhibitor, empagliflozin, resulted in reduced molecular and histological markers of kidney fibrosis and tubule damage (kidney injury molecule-1, neutrophil gelatinase-associated lipocalin) when administered with metformin or linagliptin (28, 29). SGLT2 inhibitor treatment in HK-2 cells reversed high glucose-induced inflammatory marker expression (toll-like receptor 4, NF-kB) (30). Furthermore, the EMPA-REG trial outcome provided clinical evidence that SGLT2 inhibitors reduce nephropathy incidence or progression (31). Tofogliflozin has also been investigated for preventing progression of diabetic nephropathy (32).

However, it seems that dapagliflozin and pioglitazone combination therapy involves other renoprotective mechanisms beyond glucose-lowering effects. Based on our study, although blood glucose levels were lowest in the combination group at the end of treatment, there was no statistically significant difference in blood glucose or oral glucose tolerance test between the three treatment arms. Kidney weights and morphologic changes were mostly improved in the combination group. In addition, only the combination therapy showed significantly reduced TGF-β and AGT mRNA expression. Hyperglycemia and dyslipidemia contribute to renal injury via increased oxidative stress and impaired sodium handling (33, 34). At the early onset of diabetic nephropathy, sodium reabsorption in proximal tubules is upregulated, and activated tubuloglomerular feedback increases the single nephron glomerular filtration rate, referred as hyperfiltration (35). As diabetic nephropathy progresses, GBM thickening, mesangial matrix expansion, extracellular matrix accumulation, and tubulointerstitial fibrosis appear. The increase in proximal tubular reabsorption results in tubular hypertrophy and the structural change is mediated by inflammation and growth factors, mainly TGF-β (36). Along with TGF-β activation, upregulated RAS-related components (renin and angiotensin II) bind to vascular endothelial growth factor and induce renal cell growth and extracellular matrix synthesis (37). The RAS involvement in stimulating morphogenesis in renal cells is mainly mediated through angiotensin II type 1 receptor (38). Moreover, angiotensin II mediates transcription of the TGF-β receptor gene, directly upregulating TGF-β or indirectly stimulating MCP-1 (38). The renal RAS pathway also is involved in cardiovascular disease (39), which is reflected in the cardiovascular benefit of SGLT2 inhibitors (31).

Although we demonstrated the improvement on renal morphology and renin and angiotensin gene expressions in combination therapy, we could not find any additive effect on blood glucose level. Interestingly, the body weights of the combination group were more similar to those of the dapagliflozin group than the pioglitazone group. Considering the small dose of dapagliflozin, the body weight differences between the intervention groups could be meaningful. In addition, our results showed that a small dose of dapagliflozin had equivalent efficacy to pioglitazone on glucose lowering and nephropathy prevention.

The current study provides *in vivo* and *in vitro* evidences that dapagliflozin, pioglitazone, and combination therapy attenuated diabetic nephropathy including albuminuria, renal hypertrophy, and inflammatory and fibrotic markers. In addition, this investigation demonstrated decreased renal expression of RAS components in treated mice. To our knowledge, the current study is the first to compare the renoprotective effect of SGLT2 inhibitor combination therapy to pioglitazone. Although we did not elucidate the synergistic mechanism of combination therapy, we observed a tendency toward lower profibrotic and inflammatory gene expression in the combination therapy group, which might have been due to the additive effect of those two medications. With respect to body weight, the combination therapy would be expected to provide complementary effects.

In conclusion, the current study showed that dapagliflozin, pioglitazone, and combination therapy significantly attenuated diabetic nephropathy progression, and that the renoprotective effect was magnified by combination treatment. Therefore, dapagliflozin and pioglitazone combination therapy could be an effective option to prevent diabetic nephropathy.

## Author contributions

EH designed and conducted study, collected, analyzed and interpreted data, and wrote manuscript. ES designed and conducted study, collected data, and wrote manuscript. GK and J-YL collected and analyzed data. YL designed study, analyzed and interpreted data, and wrote manuscript. B-WL and EK revised manuscript content. EH, ES, YL, and B-SC approved the final version of manuscript and take responsibility for the integrity of the data analysis.

### Conflict of interest statement

The authors declare that the research was conducted in the absence of any commercial or financial relationships that could be construed as a potential conflict of interest.
