# Supplementary material for: Combining SGLT2 Inhibition With a Thiazolidinedione Additively Attenuate the Very Early Phase of Diabetic Nephropathy Progression in Type 2 Diabetes Mellitus
Source: Front Endocrinol (Lausanne). 2018 Jul 19;9:412. doi: 10.3389/fendo.2018.00412 (PMC6060671; doi:10.3389/fendo.2018.00412)

Supplementary Table 1. Primer sequences used for RT-PCR

Human primers	Sequences (5’→3’)	GenBank reference sequences
β-actin	F-GGACTTCGAGCAAGAGATGG<br>R-AGCACTGTGTTGGCGTACAG	NM_001101.4→NP_001092.1
AGT	F-AAAGCAGCCGTTTCTCCTTG<br>R-TTCACAAACAAGCTGGTCGG	NM_001101.4→NP_001092.1
MCP-1	F-CCCCAGTCACCTGCTGTTAT<br>R-TGGAATCCTGAACCCACTTC	NM_002982.3→NP_002973.1
Renin	F-TCGTCTTTGACACTGGTTCGTCCA<br>R-AGCCACTGACTGTCCCTGTTGAAT	NM_000537.3→NP_000528.1
TGF-β	F-GGGACTATCCACCTGCAAGA<br>R-CCTCCTTGGCGTAGTAGTCG	NM_000660.6→NP_000651.3
IL-6	F-CCAGCTATGAACTCCTTCTC<br>R-GCTTGTTCCTCACATCTCTC	NM_000600.4→NP_000591.1
Mouse primers	Sequences (5’→3’)	GenBank reference sequences
Gapdh	F-TGCCTCCTGCACCACCAACT<br>r- TGCCTCCTGCACCACCAACT	NP_001256799.2→NP_001243728
Agt	F-CCTCCCGACTAGATGGACAC<br>r-AAATCCAGAGAGCGTGGGAA	NM_007428.3→NP_031454.3
Mcp-1	F- TTAAAAACCTGGATCGGAACCAA<br>r- GCATTAGCTTCAGATTTACGGGT	NM_011333.3→NP_035463.1
Renin	F- CCTCTACCTTGCTTGTGGGA<br>r- ATGCCTAGAACCCCGTCAAA	NM_031192.3→NP_112469.1
Tgf-β	F-TGACGTCACTGGAGTTGTACGG<br>r- GGTTCATGTCATGGATGGTGC	NM_021578.2→NP_067589.1

RT-PCR, real time polymerase chain reaction; AGT, angiotensinogen; MCP-1, monocyte chemotactic protein-1; TGF-β, transforming growth factor beta 1; GAPDH, glyceraldehyde-3-phosphate dehydrogenase.

Supplementary Figure 1. Glucose homeostasis in vehicle- and drug-treated *db/db* mice. (A) Measurement of blood glucose during oral glucose tolerance test of 9 week administration after 6-hr fasting in vehicle (black square, ■), pioglitazone (white circle, ○), dapagliflozin (white square, □), combination (black circle, ●), and (B) area under the curve of the oral glucose concentration. Data are means ± SEM (n = 5-8). *$p < 0.05$ vs vehicle by one-way ANOVA and Tukey's post hoc test.

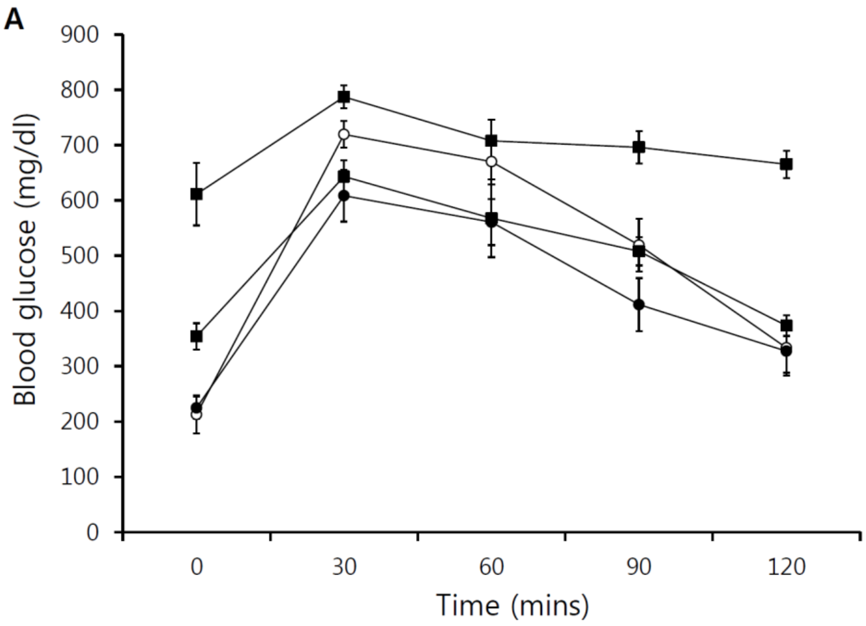

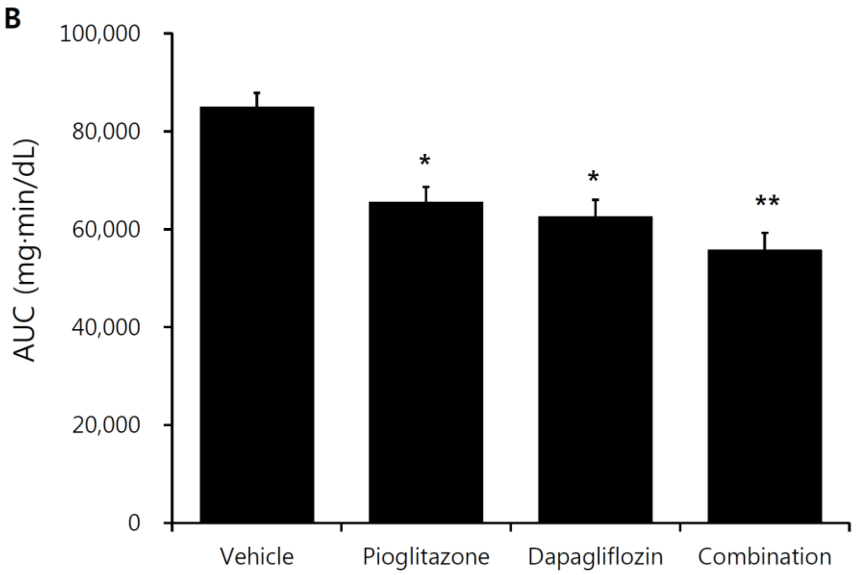

Supplementary Figure 2. Effects of pioglitazone, dapagliflozin and combination on lipid concentration. (A) Measurement of serum triglyceride and (B) serum free fatty acid after 9 weeks treatment in vehicle (PBS), pioglitazone (30 mg/kg/day), dapagliflozin (1 mg/kg/day), and combination (30 mg/kg/day of pioglitazone and 1 mg/kg/day of dapagliflozin). Data are means ± SEM (n = 5-8). **p* < 0.05 vs vehicle, ***p* < 0.001 vs vehicle by one-way ANOVA and Tukey's post hoc test.

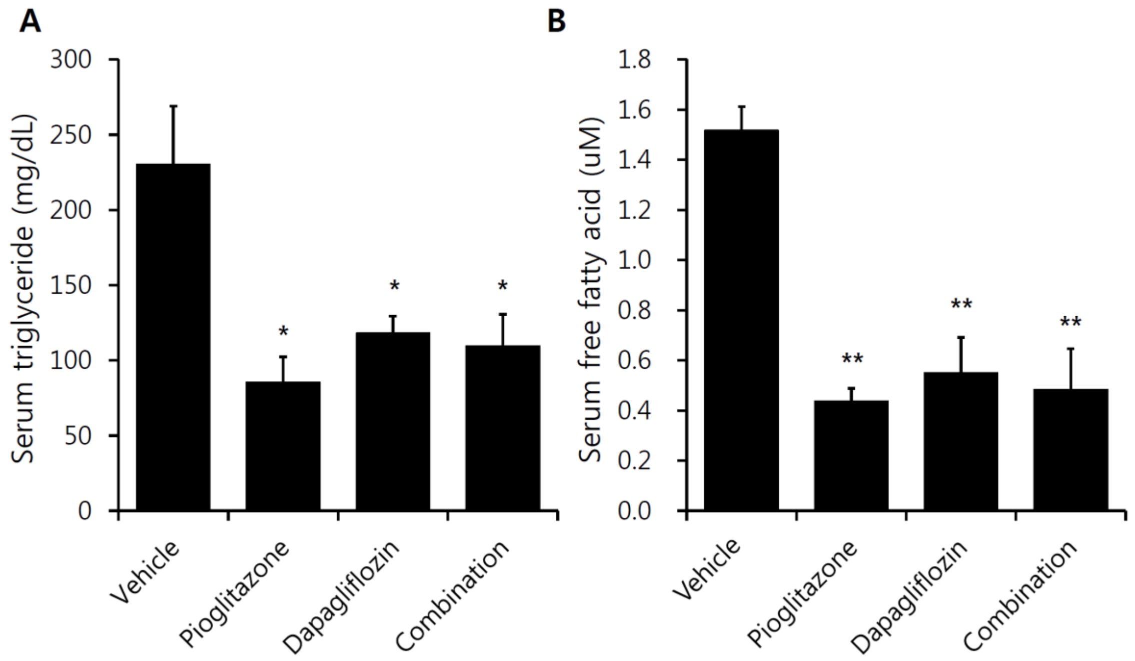

Supplementary Figure 3. Effect of pioglitazone, dapagliflozin, and combination therapy on inflammatory, profibrotic, and renin-angiotensin system-related gene expression and cell viability of HK-2 cells. HK-2 cell were exposed to either 5.5 mM glucose (normal glucose, NG), 50 mM glucose (high glucose, HG), 0.3 mM palmitic acid (PA), 10 μM pioglitazone (PIO), 10 μM dapagliflozin (DAPA), or 10 μM pioglitazone plus 10 μM dapagliflozin (COMBI). Real-time PCR for 24 hr cultured HK-2 cells for (A) transforming growth factor-β, (B) monocyte chemoattractant protein-1, (C) interleukin-6, and (D) angiotensinogen. (E) MTT assay was performed to determine cell viability in HK-2 cell. Data are means ± SEM ($n \geq 4$). *$p < 0.05$ vs high glucose and palmitic acid group, **$p < 0.001$ vs high glucose and palmitic acid group by one-way ANOVA and Tukey's post hoc test.

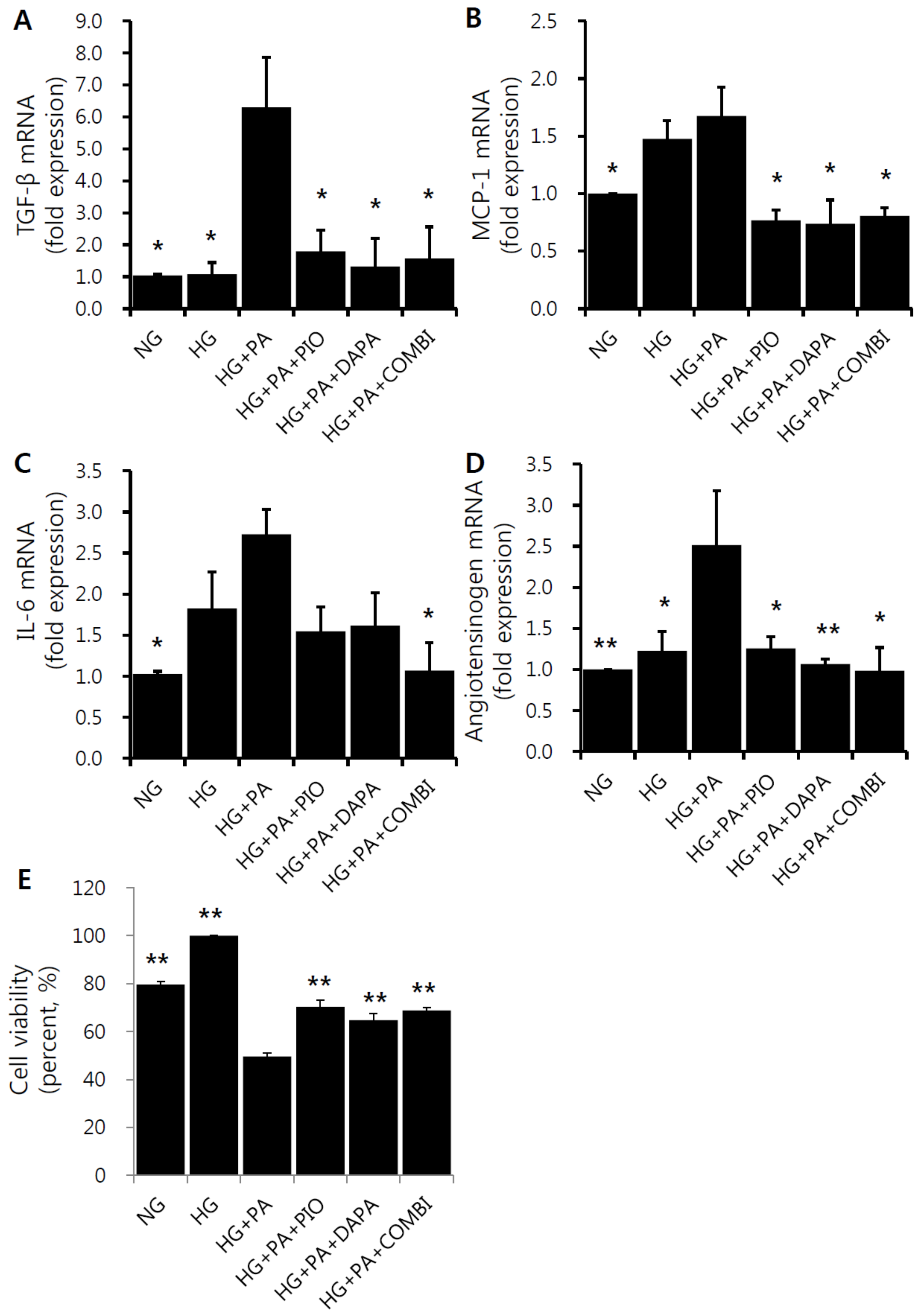
A
TGF-β mRNA (fold expression)
9.0
8.0
7.0
6.0
5.0
4.0
3.0
2.0
1.0
0.0
B
MCP-1 mRNA (fold expression)
2.5
2.0
1.5
1.0
0.5
0.0
C
IL-6 mRNA (fold expression)
3.5
3.0
2.5
2.0
1.5
1.0
0.5
0.0
D
Angiotensinogen mRNA (fold expression)
3.5
3.0
2.5
2.0
1.5
1.0
0.5
0.0
E
Cell viability (percent, %)
120
100
80
60
40
20
0
NG
HG
HG+PA
HG+PA+PIO
HG+PA+DAPA
HG+PA+COMBI

Supplementary Figure 4. Effect of pioglitazone, dapagliflozin, and combination therapy on SGLT2 protein expression in human proximal tubular cells (HK-2 cells). HK-2 cell were exposed to either 5.5 mM glucose (normal glucose, NG), 50 mM glucose (high glucose, HG), 0.3 mM palmitic acid (PA), 10 μM pioglitazone (PIO), 10 μM dapagliflozin (DAPA), or 10 μM pioglitazone plus 10 μM dapagliflozin (COMBI). Western immunoblot for 24 hr cultured HK-2 cells for (A) total cell membrane sodium glucose co-transporter 2 (SGLT2) expression and (B) quantitative analysis of SGLT2. Data are means ± SEM (n=4). The concentration of SGLT2 protein in the HK-2 membrane fraction was not different between the groups (p = 0.786).

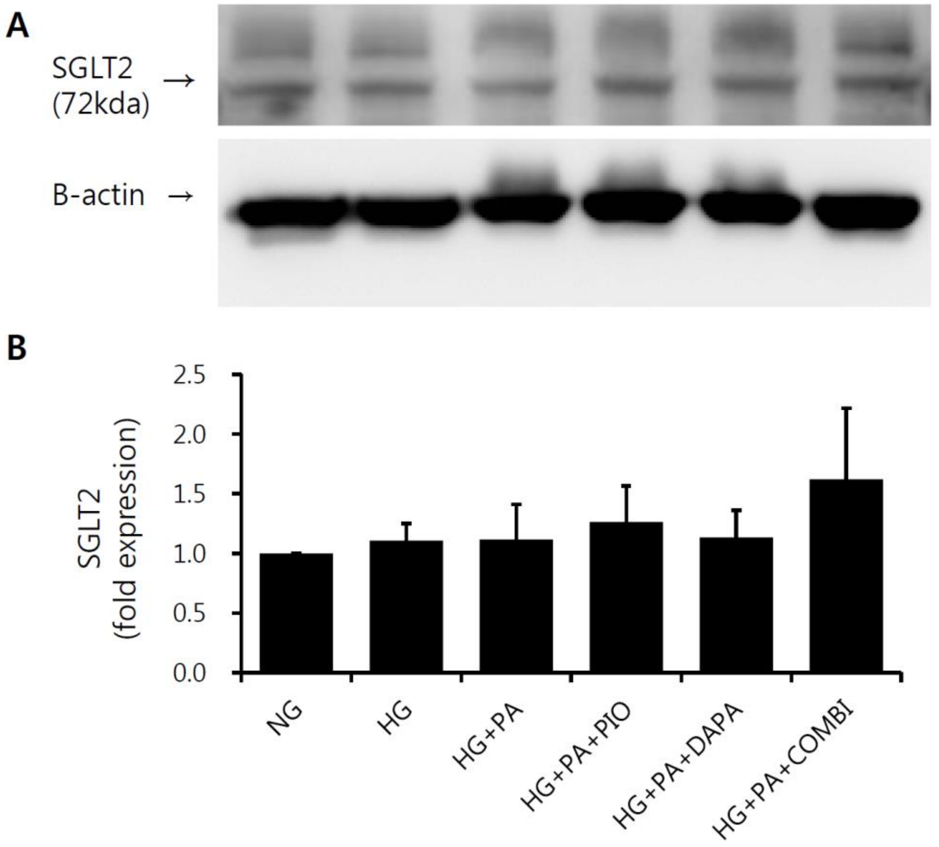

Supplement: Supplementary file 1 [file Data_Sheet_1.pdf]
